# A novel variant of NR5A1, p.R350W implicates potential interactions with unknown co-factors or ligands

**DOI:** 10.3389/fendo.2022.1033074

**Published:** 2023-01-20

**Authors:** Maki Gau, Ryota Suga, Atsushi Hijikata, Ayako Kashimada, Masatoshi Takagi, Ryuichi Nakagawa, Kei Takasawa, Tsuyoshi Shirai, Kenichi Kashimada, Tomohiro Morio

**Affiliations:** ^1^ Department of Pediatrics and Developmental Biology, Tokyo Medical and Dental University, Tokyo, Japan; ^2^ School of Medicine, Tokyo Medical and Dental University, Tokyo, Japan; ^3^ Faculty of Bioscience, Nagahama Institute of Bio-Science and Technology, Nagahama, Shiga, Japan

**Keywords:** NR5A1, NR5A2, orphan nuclear receptor, 46,XY DSD, co-factors, ligands

## Abstract

**Introduction:**

NR5A1 and NR5A2 belong to an orphan nuclear receptor group, and approximately 60% of their amino acid sequences are conserved. Transcriptional regulation of NR5A receptors depends on interactions with co-factors or unidentified ligands.

**Purpose and methods:**

We employed in vitro and in silico analysis for elucidating the pathophysiology of a novel variant in the ligand-binding domain of NR5A1, p.R350W which was identified from a 46,XY patient with atypical genitalia.

**Results:**

In the study, [1] reporter assays demonstrated that R350 is essential for NR5A1; [2] 3D model analysis predicted that R350 interacted with endogenous ligands or unknown cofactors rather than stabilizing the structure; [3] R350 is not conserved in NR5A2 but is specifically required for NR5A1; and [4] none of the 22 known missense variants of the ligand binding domain satisfied all the previous conditions [1]-[3], suggesting the unique role of R350 in NR5A1.

**Conclusion:**

Our data suggest that NR5A1 has unidentified endogenous ligands or co-activators that selectively potentiate the transcriptional function of NR5A1 *in vivo*.

## Introduction

NR5A1 and NR5A2, also known as SF-1 and LRH-1, respectively, belong to the orphan nuclear receptor group, which is characterized by a lack of identified endogenous ligands (1, 2). NR5A1 and NR5A2 share a substantially conserved sequence (approximately 60% amino acid sequence), forming the Ftz-F1 subfamily of nuclear receptors (3). Both receptors modulate cholesterol homeostasis, steroidogenesis, tissue-specific cell proliferation, and stem cell pluripotency (4, 5). Although their binding sequences are identical, both molecules exhibit different and non-overlapping effects on target organs due to their distinctive expression profiles (6). *NR5A1* (HGNC:7983) and *NR5A2* (HGNC:7984) are expressed primarily in steroidogenic tissues and in tissues of endodermal origin and gonads, respectively (7, 8). Due to its critical role in gonadal development and testicular differentiation, loss-of-function mutations in NR5A1 cause 46,XY disorders of sex development (DSD) (4, 9). While NR5A2 deletion in mice is embryonically lethal (10, 11), consequences of NR5A2 mutation in humans are still unknown (3).

The regulation of NR5A receptor transcriptional activity has been suggested to be dependent on interactions with co-factors. SHP (NR0B2) (12), DAX1 (NR0B1) (13, 14), NCOR1, NCOR2 (15), and β-catenin (16, 17) are major cofactors that can interact with NR5A1 and NR5A2. Regulation by cofactors can be either positive (co-activation) or negative (co-repression). The interactive domains in NR5A1 and NR5A2 vary depending on the type of cofactor (3).

Recent *in vitro* studies have suggested that, in addition to co-factors, unidentified ligands for NR5A receptors could regulate its activity (18, 19). Crystallography and mass spectrophotometry analyses revealed large hydrophobic pockets that were occupied by phospholipids, especially phosphatidylinositol- (3–5)-trisphosphate in both NR5As. Introducing point mutations in the ligand-binding domains such as A269, G341, L344, V348, A433, Y436, and K440 of *hNR5A1* failed to recruit co-activators and induce transcription (20, 21). Several pharmacological ligands have been developed for NR5A1 and NR5A2 (22–24), yet no reports demonstrating ligand function in mouse models or human diseases exist. Therefore, their biological relevance remains unknown.

Recently, we encountered a patient with 46,XY disorder of sex development (DSD) with a novel missense variant p.R350W in the ligand-binding domain (LBD) of NR5A1. *In vitro* analysis suggested that this variant deteriorated the transcriptional activity. Interestingly, the amino acid was not conserved between NR5A1 and NR5A2, and 3D analysis predicted that the variant would not cause a conformational change in the protein. These results suggest that arginine, the 350th amino acid of NR5A1 plays a crucial role in its interaction with co-factors or ligands, thereby providing a valuable insight into the regulation of the transcriptional activity.

## Case presentation

The patient was born full-term with a normal birth weight (40W1D, 3226 g). Although clitromegaly was noted at birth, the patient was raised as female. At 3 years of age, the patient developed a bilateral inguinal hernia that was surgically treated, but no further examination for disorder of sex development was performed. At 12 years of age, the patient exhibited pubertal development with virilization such as an increased size of the clitoris and was referred to our hospital. Chromosomal analysis revealed that the patient carried 46,XY genotype with *SRY*, and endocrinological tests suggested that the gonads had testicular function (Testosterone: 4.96 ng/mL, Estradiol: <5 pg/mL), although gonadotropin levels were elevated (LH:14.7 mIU/mL, FSH:70.7 mIU/mL). The patient showed no signs or symptoms of adrenal insufficiency. The patient was diagnosed with 46,XY DSD, and genetic analysis revealed a missense variant (NM_004959.4 c.1048 C>T, NP_004950.2 p. Arg350Trp [RefSeq version5], rs754336683) in the NR5A1 gene. We did not perform genetic analysis of the parents, but they did not show any signs or symptoms of the disorder of sex development. The frequency of the variant is low, 0 in gnomAD (https://gnomad.broadinstitute.org/gene/ENSG00000136931?dataset=gnomad_r3), and according to the American College of Medical Genetics guidelines, the variant was classified as highly pathogenic. Registration of the variant in our case resulted in a MAF (minor allele frequency) of 0.00005665 in the East Asian population (https://gnomad.broadinstitute.org/variant/9-127253450-G-A?dataset=gnomad_r2_1).

## Materials and methods

The study was approved by the Institutional Review Board Committee at Tokyo Medical and Dental University and written informed consent was obtained from the parents (G2000-103). We confirmed that all methods were performed in accordance with the relevant guidelines and regulations.

### Mutation analysis

Genomic DNA was obtained from the lymphocytes of the patient, and DNA analysis was conducted using the TruSightTM One sequencing panel, #FC-141-1006 (Illumina, CA, USA). The known causative genes for 46,XY DSD, such as *AR* (HGNC:644), *HSD17B3* (HGNC:5212), *HSD3B2* (HGNC:5218), *NR5A1*, *SRD5A2* (HGNC:11285), *WT1* (HGNC:12796), *ANOS1* (HGNC:6211), *CHD7* (HGNC:20626), *FGF8* (HGNC:3686), *FGFR1* (HGNC:3688), *and SRY* (HGNC:11311), were screened, and a missense variant c.1048 C>T was identified in the NR5A1 gene, which was further confirmed by Sanger sequencing.

### Plasmids

The expression vector for wild-type human *NR5A1* was purchased from the Kazusa DNA Research Institute (Kisarazu, Chiba, Japan). The expression vectors for mutant *NR5A1*, R350W, R350L, R350H, A260V, C283R, and Q460R, were generated by mutagenesis using a QuikChange II Site-Directed Mutagenesis Kit (Agilent, CA, USA). Reporter vectors containing the *SOX9* (HGNC:11204) testis enhancer sequence (hTES) and the promoter of *CYP17* (HGNC:2593) were kindly gifted by V. Harley (Hudson Institute of Medical Research) (25) and JS. Richards (Baylor College of Medicine), respectively.

### Luciferase assay

The transcriptional activities of the wild-type and the missense variants of NR5A1 on hTES and the *CYP17* promoter were determined by dual luciferase reporter assays. CHO cells were prepared in 24-well plates (1.0 × 10^5^ cells/well). *NR5A1* (100 ng) of the wild-type or the missense variant expression plasmids or the pcDNA3 empty plasmids were transiently introduced with 200 ng of the reporter plasmids (hTES or the *CYP17* promoter). Relative luciferase activity was measured 48 h after transfection using the Dual-Luciferase Reporter Assay System (Promega, MD, USA). Samples were measured in triplicates in each assay, and all the assays were independently repeated three to four times.

### Western blotting

The *NR5A1* expression plasmids of the wild-type or the missense variants were introduced into CHO cells, and whole-cell lysates were collected using lysis buffer containing 50 mM Tris-HCl (pH 8.0), 50 mM NaCl, 1 mM EDTA, and 1% SDS (26). After SDS-PAGE, immunoblotting was performed with anti-Ad4BP/SF-1 antiserum (1:1000, a kind gift from K. Morohashi Lab) and anti-β-actin antibody (1:10000, A1978, Sigma-Aldrich, St. Louis, MO, USA). Anti-rabbit IgG (1:5000, GE Healthcare, UK) and anti-mouse IgG (1:10000, GE Healthcare, UK) were used as secondary antibodies.

### In silico analysis of missense mutations on a three-dimensional structure model

Mutated residues were mapped to the crystal structure of human NR5A1 (PDB code: 4qjr). The effects of each missense variant on thermal stability were evaluated using FoldX 5.0 software (26). Putative interfaces of molecular interactions were predicted using the SPPIDER web server(http://sppider.cchmc.org/) (27). Supramolecular structural modeling of NR5A1 and its interacting molecules was performed by superimposing the structure of NR5A1 in complex structures according to previously described methods (28). Prediction analysis was performed according to the guidelines of Vihinen M (29). REVEL scores for the predicted pathogenicity of missense variants were estimated using a computational method (30).

### Immunofluorescence analysis

We transiently introduced expression vector of wild type or R350W NR5A1 into CHO cells and performed immunofluorescence analysis as previously described (15, 31). The rabbit anti-NR5A1 (Ad4BP) antibody (kindly provided by K Morohashi) was used at a 1:500 dilution, and the goat Texas-Red dye-conjugated anti-rabbit antibody (#111-076-047, Jackson ImmunoResearch, PA, USA) was used as the secondary antibody at 1:100 dilution. The slides were mounted with Vectashield mounting medium (H-1200; Vector Laboratories, Burlingame, CA, USA), allowing for nuclear 4,6-diamidino-2-phenylindole (DAPI) staining. Images were obtained using a confocal microscope, TCS-SP8 (Leica microsystems, Germany).

## Results

### R350 was essential for NR5A1 function *in vitro*

**Figure 1:**
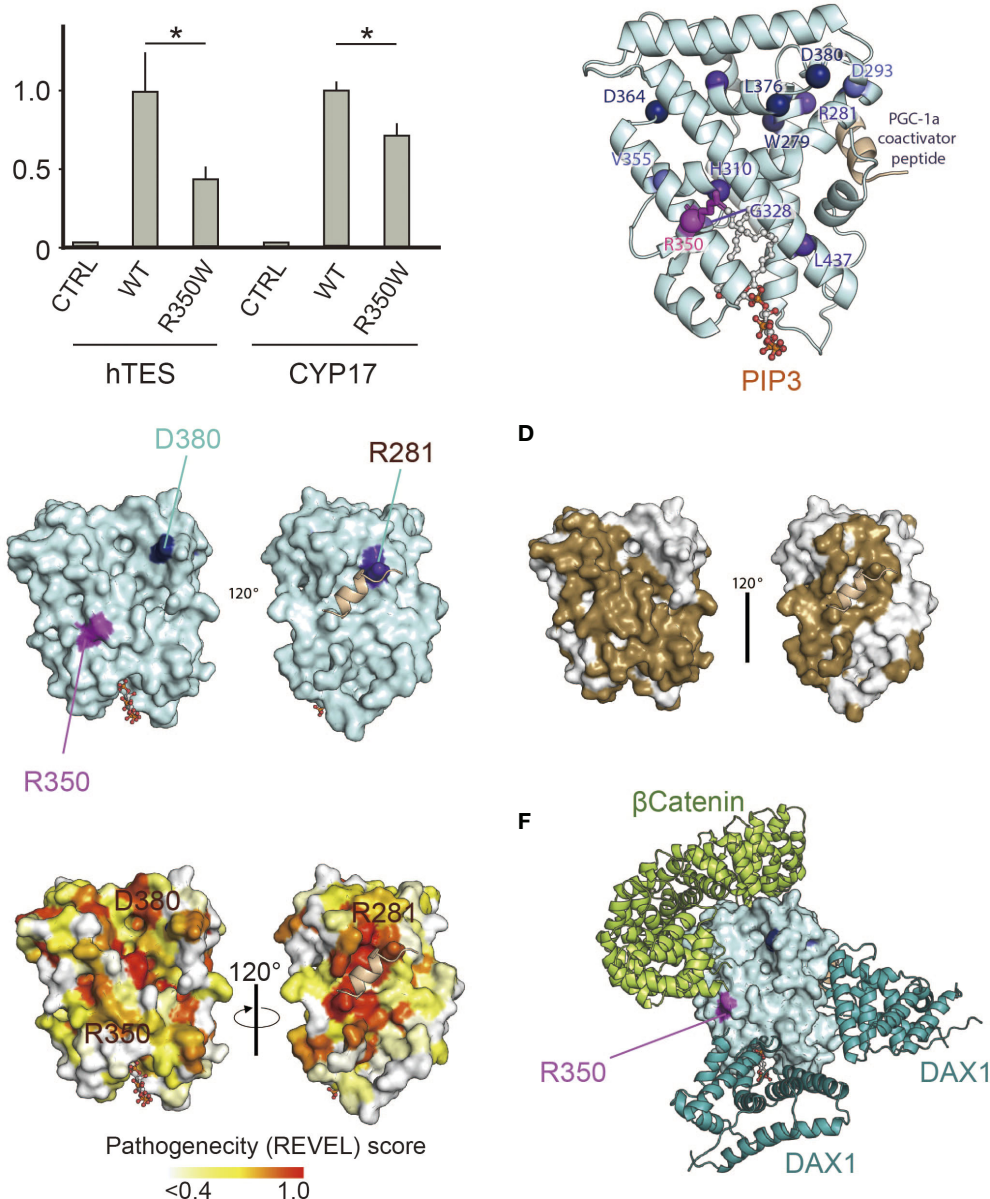
**(A)** p.R350W variant of NR5A1 revealed significantly impaired translational activities. Reporter assay of the wild type and R350W for hTES, the human testicular enhancer for *Sox9*, and the promoter of *CYP17*. Data sets represent activation of the reporters relative to wild type, whose value was adjusted to 1.0. The mean and SD of three to five biological replicates measured was calculated. Asterisks indicate statistical significance (*, P <0.05). Unpaired Student’s t test was used to demonstrate statistically significant difference between the given sample and the wild type. **(B)** 3D model analysis of LBD in NR5A1, indicating that R350W was on the helix7. **(C)** R350 (purple) was located on the surface of LBD in NR5A1. **(D)** The 3D model analysis by SPPIDER server (http://sppider.cchmc.org/) predicted the surface area of the LBD to interact with other molecules (Brown). The location of R350 indicated in Fig. 1C is covered by the brown area. **(E)** The REVEL pathogenicity score was elevated in R350 and its surrounding area. **(F)** Known co-factors that bind to the LBD of NR5A1, β-catenin and DAX1(NR0B1) were not predicted to interact with R350 directly in the 3D model of the predicted protein complexes.

The clinical phenotypes of the patient suggested that R350 is essential for NR5A1 function *in vivo*. Reporter analysis revealed that p.R350W significantly impaired the transcriptional activities of hTES and the *CYP17* promoter, which are thought to play major roles in Sertoli and Leydig cell differentiation (**Figure 1A**). This assay confirmed the essential role of R350 in NR5A1 *in vitro*.

### The predicted role of R350 was interacting with other molecules

**Figure 2:**
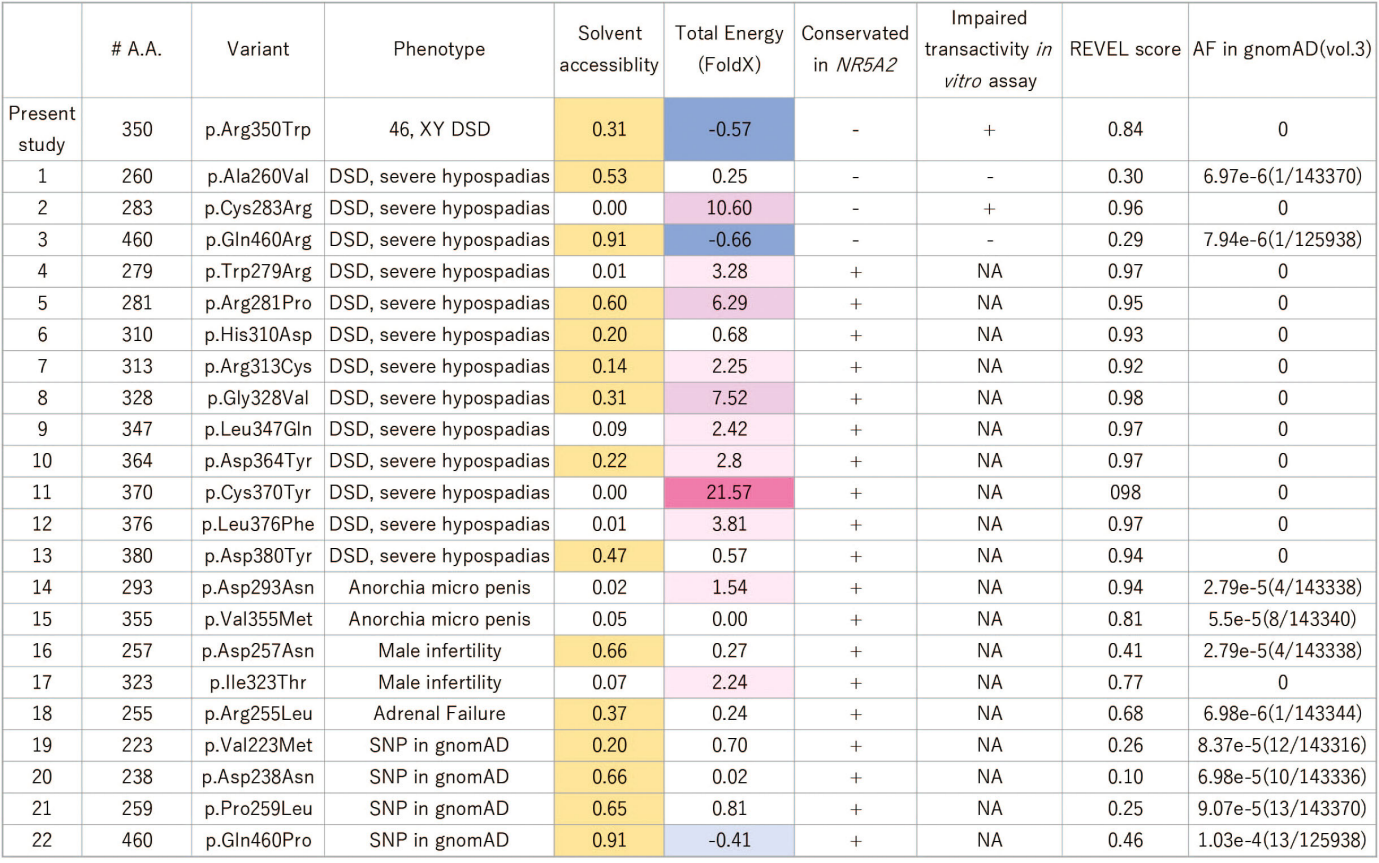
Summary of the reported missense variants on the NR5A1 LBD. Yellow colored amino acids are predicted to be located outside surface. In the LBD of NR5A1, 22 missense variants have been identified in patients with 46,XY DSD to date. Of these 22 amino acid residues, 19 were conserved in NR5A2, and only three, p.A260V, p.C283R, and p.Q460R, were not conserved. The functional analyses showed that p.A260V and p.Q460R were not deleterious variants. The thermal stability analysis (FoldX), indicated that p.R350W would not destabilize LBD structure while p. C283R variant would. The specificity of the REVEL score at 0.5 and 0.75 for pathogenic are 89.1% and 95%, respectively (31). DSD, Disorder of Sex Development; NA, not available.

*In silico* 3D analysis revealed that R350 was on helix7, which is a major component of the ligand-binding pocket (**Figure 1B**), and R350 was located external of the predicted potential molecular interface (**Figures 1C, D**, **2**). REVEL scores were elevated in p.R350W and its surrounding areas suggesting the importance of R350 and its neighboring amino acids for NR5A1 function (**Figure 1E**). The computational software FoldX predicted that p.R350W would increase the thermal stability of NR5A1, suggesting that p.R350W would not destabilize the LBD structure. These results indicate that p.R350W is a unique missense mutation, as most of the previously reported missense pathological variants in the LBD decreased stability (**Figure 2**). Further analysis of the known complex structures of the NR5A1-LBD and its co-factors, β-catenin and DAX1, showed that R350 was not in direct contact, although it was in close proximity to these molecules (**Figure 1F**). Taken together, the predicted role of R350 would be a part of the interaction sites for other molecules, that is, an endogenous ligand or an unknown co-factor, rather than being a stabilizing factor for the LBD structure.

### Function of the 350^th^ amino acid residue in NR5A1 was distinctive from that in NR5A2

**Figure 3:**
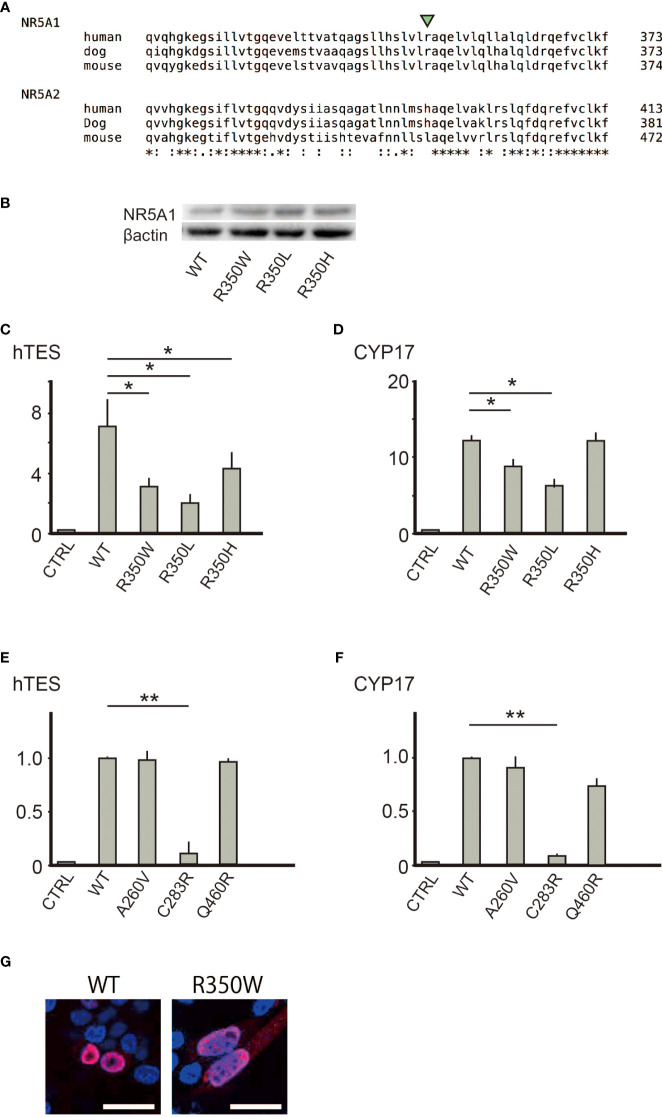
**(A)** R350 is conserved among NR5A1 of various species, but not in NR5A2. **(B)** Substitution of the 350^th^ arginine with tryptophan, leucine, or histidine did not affect the protein expression levels. **(C, D)** Substitution of the 350^th^ arginine with leucine or histidine reduced the trans-activating ability in hTES and the *CYP17* promoter. Asterisks indicate statistical significance (*, P <0.05). **(E, F)** Of the three missense variants (p.A260V, p. C283R, and p.Q460R) suggested to cause 46,XY DSD, only p.C283R reduced the trans-activating ability in hTES and the *CYP17* promoter. Data sets represent activation of the reporters relative to wild-type whose value was adjusted to 1.0. The mean and SD values of three to five biological replicates measured were calculated. Asterisks indicate statistical significance (**, P <0.001). Unpaired Student’s t-test was used to demonstrate statistically significant difference between the given sample and the control. **(G)** Immunofluorescence of transiently transfected CHO cells with the wild type or R350W NR5A1 expression vectors. The nuclear localization was identical between wild type and R350W (red) White bar indicate 25µm.

Despite the conservation of R350 in NR5A1 among species, the equivalent amino acid residue was altered to leucine or histidine in NR5A2 (**Figure 3A**), suggesting that an unknown molecule would exclusively interact with NR5A1 through R350. To verify the exclusivity of the potential interaction, we assessed whether the function of R350 could be replaced with the corresponding amino acid residues in NR5A2, leucine or histidine, and carried out a reporter assay for NR5A1 whose R350 was substituted with leucine (p.R350L) or histidine (p.R350H). Although the protein expression of p.R350L and p.R350H (**Figure 3B**) was not affected, the transcriptional activities of p.R350L and p.R350H were significantly impaired (**Figures 3C, D**). Thus, NR5A1 specifically requires arginine as the 350^th^ amino acid residue, and the function of the 350^th^ amino acid residue in NR5A1 is distinct from the corresponding amino acid residue in NR5A2. However, nuclear localization of R350W was not affected (**Figure 3G**).

### Comparison to the other missense pathogenic variants on the LBD revealed the unique function of the R350 role in NR5A1

In the LBD of NR5A1 of patients with 46,XY DSD, 22 missense variants have been identified, suggesting that these 22 amino acids play essential roles in the LBD of NR5A1 *in vivo*. Of these 22 amino acid residues, 19 were conserved between NR5A1 and NR5A2 while three, p.A260V, p.C283R, and p.Q460R, were not conserved (**Figure 2**). The reporter assay revealed that the trans-activating activity was impaired in the variant p. C283R, but not in p. A260V or p. Q460R (**Figures 3E, F**), indicating that p. A260V and p.Q460R are not pathological variants. Thus, in addition to p.R350W, p. C283R is the only pathological missense variant whose substituted amino acid residue is not conserved in NR5A2. During thermal stability analysis, the LBD with the p. C283R variant was destabilized (**Figure 2**), and the major role of C283 was to stabilize the structure of the LBD rather than to interact with other molecules, co-factors, or ligands.

## Discussion/Conclusion

R350 has an essential role in NR5A1, as we have proven *in vivo* (46,XY DSD) and *in vitro* (reporter assays). Our present study suggests that there is an endogenous ligand or a co-activator that selectively potentiates the transcriptional function of NR5A1, but not that of NR5A2, *in vivo*. The profoundly impaired testicular function in our case with severely under-virilized external genitalia indicated that R350 in the LBD of NR5A1 plays an essential role in testicular development in humans, which was confirmed by our *in vitro* reporter assay. *In silico* 3D analysis predicted that the major function of R350 was interacting with other molecules, such as co-factors and endogenous ligands, rather than maintaining the structure of NR5A1. However, R350 was not conserved in NR5A2. Furthermore, NR5A1 exclusively requires arginine as the 350^th^ amino acid residue, as substitution of R350 with the corresponding histidine/leucine residues in NR5A2 did not compensate for the transcriptional activity of NR5A1.

Although identifying the molecule interacting with R350 is beyond the scope of this study, we presume that known co-factors are not likely to be involved in the interaction with NR5A1 through R350. The only known co-factor that activates NR5A1 trans-activity is β-catenin (3), which is unlikely to be involved in the pathophysiology of severely impaired testicular development. During gonadal development, β-catenin acts in favor of ovarian development by suppressing key molecules for testicular development, such as FGF9 (32). In this biological context, β-catenin presumably counteracts NR5A1 function rather than activating it. Indeed, supramolecular complex models of NR5A1 predicted that R350 did not mediate the interaction with β-catenin. Furthermore, all known cofactors have been suggested to interact with both NR5A1 and NR5A2 or NR5A2 alone (3). In other words, co-activators whose association is limited to NR5A1 have not yet been reported. Identifying novel co-factors of NR5A1 will advance our understanding of its function.

Another implication of our data is the possible presence of an endogenous ligand *in vivo*. Although the unaffected nuclear localization of R350W suggests that its binding ability was maintained, R350 may have essential roles in ligand-dependent conformational changes of NR5A1. To the best of our knowledge, no data using mouse models or human diseases have revealed that NR5A1 requires an endogenous ligand for its transcriptional activity. Furthermore, our data suggest the existence of an endogenous ligand selective for NR5A1, as the equivalent amino acid residue of NR5A2 did not restore NR5A1 function. Recently, artificial ligands that can bind to either NR5A1 or NR5A2 have been developed. Low molecular weight compounds containing cis-bicyclo [3.3.0]oct-2-ene in their core structure selectively increases NR5A1 activity (23), whereas dilauryol-phosphatidyl-choline (DLPC) activates NR5A2 (22). These findings indicate that NR5A1 and NR5A2 have similar structures but require different ligands; however, it is not clear whether there are active endogenous ligands for either NR5A receptor *in vivo* (33). Our observations support the *in vitro* observation of artificial ligands, thereby shedding new light on the unidentified ligands of NR5A1.

It is intriguing that the trans-activity of p.R350H varied depending on the reporters; the trans-activity was maintained on the *CYP17* promoter, but not on the hTES promoter. The interaction between cofactors and NR5A1/NR5A2 is presumed to be controlled by ligands, and cofactors of NR5A1/NR5A2 are known to regulate its transcriptional activity in a context-specific manner (3). Our data would be an example to show the complexity of the interaction between NR5A1/NR5A2 and co-factors/ligands.

The present study has some limitations. The parents of the patient did not allow us to perform a familial analysis, which could have provided further insights. Using our 3D model analysis, it was difficult to completely exclude the possibility that p.R350W would indirectly interfere with the allosteric regulation of the known co-factors for ligand binding. Our analysis is based on a single-case study, and further case studies are required to elucidate the precise function of LBD, especially regarding its interaction with other molecules. Nevertheless, the 46,XY DSD case with p.R350W provided intriguing insights into possible molecules, co-factors, or endogenous ligands interacting with NR5A1.

NR5A1 has been reported to play a role in organ development and oncogenesis (3, 34). We envisage that, in addition to identifying the pathophysiology of DSD, an improved understanding of the molecules interacting with NR5A1 may provide novel insights, leading to the development of valuable therapeutic options for malignant diseases.

## Data availability statement

The data presented in the study are deposited in the ClinVar repository, accession number SCV002099761.

## Ethics statement

The studies involving human participants were reviewed and approved by the Institutional Review Board Committee at Tokyo Medical and Dental University. Written informed consent to participate in this study was provided by the participants’ legal guardian/next of kin.

## Author contributions

KK, MG, and KT contributed to the conception and design of this study, acquisition of data, and analysis and interpretation of the data. All experiments were performed by MG, RS, AH, AK, MT, RN, KT, and TS. The manuscript was drafted by KK and MG and critically revised by AH and TM. All authors have read and approved the final manuscript. All authors contributed to the article and approved the submitted version.
